# The Vital Illusion: Cone-Beam Computed Tomography (CBCT)-Tracked Evolution of Periapical Cemento-Osseous Dysplasia

**DOI:** 10.7759/cureus.113758

**Published:** 2026-07-31

**Authors:** Serkan Ucmak, Enver Faydali

**Affiliations:** 1 Department of Endodontics, Faculty of Dentistry, Selçuk University, Konya, TUR

**Keywords:** cemento-osseous dysplasia, cone-beam computed tomography, diagnosis, differential, fibro-osseous lesion, pulp vitality

## Abstract

Periapical cemento-osseous dysplasia (PCOD) is a benign fibro-osseous lesion that, in its early osteolytic stage, can mimic inflammatory periapical disease and prompt unnecessary endodontic treatment. This report documents its radiographic evolution in a 44-year-old woman referred for endodontic evaluation of the mandibular left canine and premolars. A previous panoramic radiograph obtained as a baseline was unremarkable. At initial presentation, panoramic and cone-beam computed tomography (CBCT) imaging revealed periapical radiolucencies around the apices of the canine, first, and second premolars, with a central radiopacity beneath the canine, consistent with transition to the mixed stage. All involved teeth were asymptomatic and responded normally to cold and electric pulp testing. No treatment was performed. At 13.5-month follow-up, the central radiopacity had enlarged while the teeth remained vital. Recognizing PCOD and confirming pulp vitality prevent misdiagnosis and overtreatment; periodic clinical and radiographic monitoring is the appropriate management.

## Introduction

Cemento-osseous dysplasia (COD) is the most common benign fibro-osseous lesion of the tooth-bearing jaws [1]. It is a reactive, non-neoplastic process in which the normal bone around the tooth apex is gradually replaced by fibrous tissue containing disorganized bone-like and cementum-like material, and it is thought to arise from cells of the periodontal ligament [2,3]. The current World Health Organization classification recognizes the following three clinical forms according to the extent of jaw involvement: periapical, focal, and florid [1,3]. The periapical form (periapical cemento-osseous dysplasia {PCOD}) is centered on the apices of the mandibular anterior teeth and, less often, extends to the canine and premolar region; it occurs most frequently in middle-aged women [3-6].

PCOD passes through three radiographic stages. In the early, osteolytic stage, the lesion appears as a periapical radiolucency and can be mistaken for a periapical granuloma or cyst. In the intermediate, mixed stage, radiopaque foci of mineralized tissue develop within the radiolucency. In the mature, cementoblastic stage, the lesion becomes densely radiopaque, often outlined by a thin radiolucent rim [4,7-9]. The single most useful feature that separates PCOD from inflammatory periapical disease is that the associated teeth remain vital, because the lesion does not originate in the pulp; consequently, these teeth require no endodontic treatment [7,10]. Overlooking this point may lead to unnecessary root canal treatment or even extraction.

Because the lesions overlap the roots on two-dimensional images, panoramic and periapical films may not reveal the true nature and extent of the process, and preoperative three-dimensional imaging has been shown to be of value in guiding the diagnosis and treatment of complex periapical and root canal anatomy [11]. Cone-beam computed tomography (CBCT) provides a superimposition-free three-dimensional view and is more accurate than plain films for evaluating periapical bone [12-14]. Reports that follow a single lesion from a normal baseline through successive stages are uncommon [7]. This report presents a case of PCOD in which an unremarkable baseline radiograph, later panoramic and CBCT imaging, and short-term follow-up together document the transition into the mixed stage, and it emphasizes the role of pulp testing in avoiding overtreatment.

## Case presentation

Chief complaint and history

A 44-year-old woman was referred from the oral surgery clinic for endodontic evaluation of the mandibular left canine and premolars after a radiographic abnormality was noted in that region. She was systemically healthy and took no regular medication. She had never had any pain, swelling, or other complaint related to this region at any time. Written informed consent for evaluation and for the publication of clinical and radiographic images was obtained.

Clinical examination

Intraoral examination was unremarkable. The mandibular left canine (tooth #33), first premolar (tooth #34), and second premolar (tooth #35) were sound, with no significant caries or restorations, and were not tender to percussion or palpation; periodontal probing depths were within normal limits. All three teeth responded within normal limits to cold testing and to electric pulp testing, indicating vital pulps.

Radiographic examination

**Figure 1:**
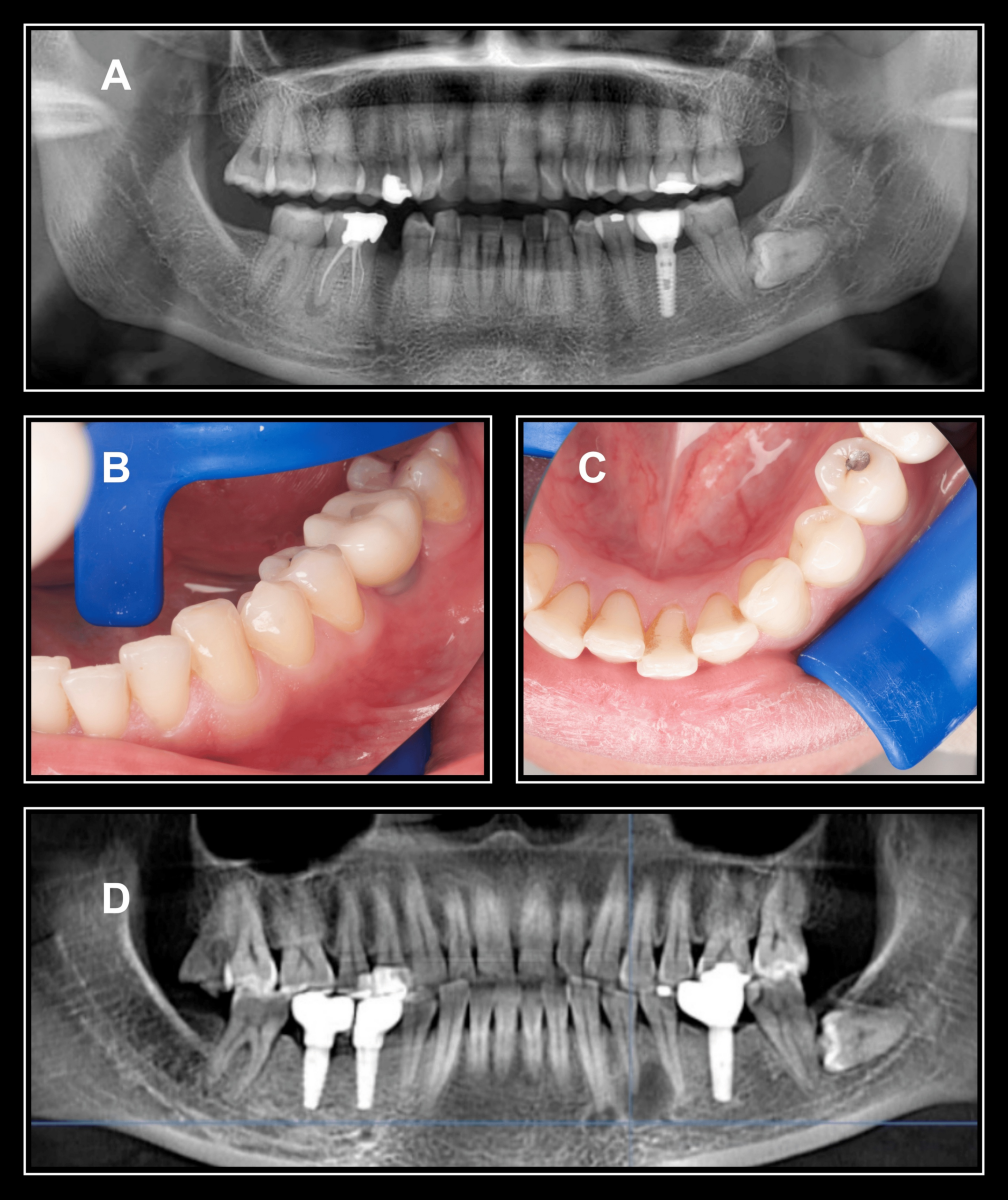
Radiographic and clinical evolution of periapical cemento-osseous dysplasia in the mandibular left canine and premolar region. (A) Panoramic radiograph obtained at baseline showing no periapical pathology in the mandibular left canine and premolar region (baseline). (B, C) Intraoral photographs of the left mandibular quadrant obtained at initial presentation. (D) Panoramic radiograph obtained at initial presentation, showing periapical radiolucencies around the apices of teeth #33 to #35.

A panoramic radiograph obtained previously for an unrelated purpose showed no periapical pathology in this region and served as a baseline (Figure 1, panel A). At initial presentation, a new panoramic radiograph and intraoral photographs were obtained as part of the referral work-up (Figure 1, panels B-D).

**Figure 2:**
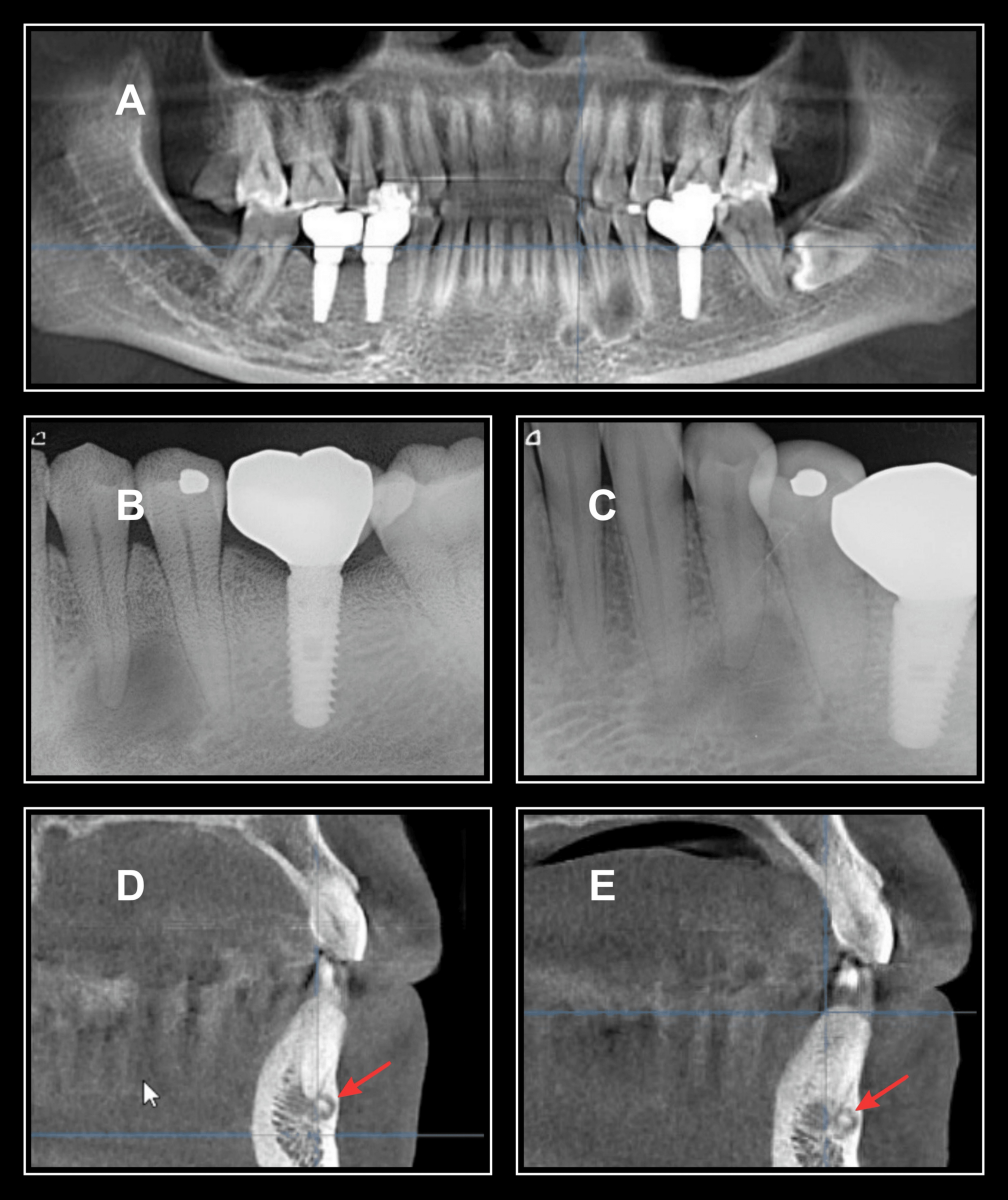
Radiographic and CBCT progression of the periapical lesion at the mandibular left canine apex over the follow-up period. (A) Panoramic radiograph obtained at follow-up. (B) Periapical radiograph obtained at initial presentation. (C) Periapical radiograph obtained at follow-up. (D) Sagittal cone-beam computed tomography (CBCT) section (initial presentation) showing a periapical radiolucency at the apex of the mandibular left canine with a central radiopacity. (E) Sagittal CBCT section (follow-up) showing the periapical radiolucency with a central radiopacity that has increased compared with the previous year. Red arrows indicate the central radiopacity.

The panoramic image now showed radiolucent areas surrounding the apices of teeth #33 to #35. Because the two-dimensional panoramic and periapical images could not fully characterize the nature and extent of the periapical changes, and to exclude inflammatory periapical disease before any endodontic intervention, a limited field-of-view CBCT scan was performed in accordance with radiation protection principles and current guidelines on the justified use of CBCT in endodontics. The CBCT examination was performed using an Instrumentarium Dental unit (Tuusula, Finland: PaloDEx Group Oy) with a limited field of view at 89 kVp and 8 mA. The panoramic radiograph, periapical radiographs, and sagittal CBCT sections showed well-defined periapical radiolucencies around the apices of the canines and premolars, with a discrete radiopaque focus at the center of the radiolucency at the apex of the canine (Figure 2, panels A, B, D).

No root resorption or tooth displacement was observed, and loss of the lamina dura was confined to the involved apices. Considering the vital pulps, the multi-tooth periapical distribution, the appearance of a central radiopacity within a previously radiolucent area, and the unremarkable baseline examination, the findings were interpreted as periapical cemento-osseous dysplasia in transition from the osteolytic to the mixed stage.

Management and follow-up

Because the teeth were vital and the patient had no symptoms, no endodontic or surgical treatment was performed. The patient was placed on periodic clinical and radiographic review and was advised to maintain good oral hygiene and to attend routine dental checks. At review approximately 13.5 months later, the patient still had no complaints of any kind, as had been the case from the start. Vitality testing was repeated as follows: cold testing and electric pulp testing were applied to teeth #33, #34, and #35, and all three teeth gave a positive response, confirming that the pulps were still vital. On the panoramic, periapical, and CBCT imaging, the radiopacity at the center of the radiolucency at the canine apex had increased compared with the initial presentation, and the lesion as a whole was shifting from a mainly radiolucent appearance toward a more radiopaque one. These changes were in keeping with continued maturation toward the mixed and cementoblastic stages (Figure 2, panels C, E) (Table 1).

**Table 1:** Timeline of imaging, pulp vitality findings, radiographic changes, and management. Summary of the imaging modalities, pulp vitality testing, radiographic findings, and clinical management at each time point. CBCT: cone-beam computed tomography; EPT: electric pulp testing

Date	Diagnostic imaging	Pulp vitality	Radiographic finding	Management
Baseline	Panoramic	Not tested	No periapical pathology (baseline)	None
Initial presentation	Panoramic, periapical, CBCT	Vital (cold and EPT positive)	Periapical radiolucencies at teeth #33-#35; central radiopacity at canine apex (osteolytic-to-mixed transition)	Clinical observation
13.5-month follow-up	Panoramic, periapical, CBCT	Vital (cold and EPT positive)	Increased central radiopacity; shift toward mixed/radiopaque appearance	Continued follow-up

## Discussion

The most important step in reaching the diagnosis was pulp testing. Because cemento-osseous dysplasia (COD) originates in the periodontal ligament rather than the pulp, the associated teeth stay vital, which distinguishes PCOD from a periapical granuloma, cyst, or abscess, in which the offending tooth is non-vital [7,10]. In this patient, all three teeth responded normally to cold and electric testing, so an inflammatory apical lesion was effectively excluded and endodontic treatment was avoided. Preserving and objectively confirming pulp vitality, whether through diagnostic testing as in this case or through vitality-preserving treatment approaches, remains a central goal of conservative endodontic practice [15]. The periapical form favors the mandibular anterior region and, as here, may extend to the canine and premolar area, and it is most common in middle-aged women, which was the case for this patient [3,4].

A further feature of this case is the documented progression of the lesion. The baseline panoramic radiograph showed no change in the region and provided a normal baseline. Because the baseline imaging was normal, it is evident that the dysplasia developed after the baseline examination; however, the exact time of onset cannot be determined. Four years later, periapical radiolucencies had appeared, and a small radiopacity had formed at the center of the lesion beneath the canine, which is the hallmark of the transition from the osteolytic to the mixed stage. A periapical cyst or granuloma was excluded by the maintained pulp vitality of all involved teeth, a cementoblastoma by the lack of attachment to a single root and the absence of a radiolucent rim around a tooth-bound mass, and idiopathic osteosclerosis by the presence of a peripheral radiolucent zone and the documented progression through radiographic stages. The radiographic and CBCT interpretation was independently reviewed and confirmed by an oral and maxillofacial radiologist. At the 13.5-month review, this central radiopacity had grown larger, and the lesion was gradually shifting from a radiolucent to a more radiopaque appearance, in keeping with continued mineralization toward the mature cementoblastic stage. PCOD is known to develop slowly over many years, and reports that follow the same lesion from a normal baseline through successive stages are uncommon [4,7]. Well-documented longitudinal case reports of this kind continue to form a valuable part of the endodontic literature, including in this journal [16]. This progression both supported the diagnosis and showed the natural course of the disease.

Because the lesions overlap the roots on two-dimensional films, their true extent and internal structure can be difficult to judge on panoramic and periapical images. CBCT provided a three-dimensional view that confirmed the periapical location, the intact adjacent cortical bone, and the central radiopacity within the radiolucency, and it helped exclude other periapical and fibro-osseous entities [12,13]. Recognized radiographic clues for COD include a radiolucent rim, a mixed internal pattern, dense cementum-like radiopacities, and loss of the lamina dura at the involved apex with a preserved periodontal ligament space elsewhere [8-10]. Caution is nonetheless required, because an osteolytic-stage lesion can resemble a periapical cyst, whereas a mature radiopaque lesion can resemble a cementoblastoma or idiopathic osteosclerosis; the combination of vital teeth, a multi-tooth periapical distribution, and typical staging is what points to PCOD [17].

Management of asymptomatic PCOD is conservative. Neither endodontic treatment nor surgery is indicated, and biopsy is generally avoided because the sclerotic, poorly vascularized bone heals slowly and is prone to infection once opened [3,6,7]. Periodic clinical and radiographic follow-up, supported by good oral hygiene to keep the teeth sound, is the accepted approach [4,5,7]. This patient was therefore monitored rather than treated. The main limitation of this report is its short follow-up; the lesion should be observed over the long term for any increase in size, development of symptoms, or secondary infection, at which point further assessment would be warranted. A single case cannot be generalized, but it reinforces two practical points: test the pulp before treating a periapical radiolucency, and consider PCOD when vital teeth carry apical lesions that change in a staged, mixed pattern over time.

## Conclusions

Periapical cemento-osseous dysplasia should be considered whenever vital teeth show periapical lesions, particularly in the mandible of a middle-aged woman. In this case, an unremarkable baseline examination, later panoramic and CBCT imaging, and short-term follow-up together documented the change from the osteolytic to the mixed stage, while pulp testing confirmed that the teeth were vital. Recognizing this pattern prevented misdiagnosis and unnecessary endodontic or surgical intervention. Because the lesion is benign and slowly progressive, periodic clinical and radiographic monitoring, rather than treatment, is the appropriate management for a patient without symptoms.
